# Non-invasive identification of steatohepatitis in patients with MASLD using a sterol and lipidomic signature

**DOI:** 10.1016/j.jlr.2025.100845

**Published:** 2025-06-20

**Authors:** Ratna Budhi Pebriana, Ting Chen, Rico J.E. Derks, Niek Blomberg, Aldo Grefhorst, Yassene Mohammed, Max Nieuwdorp, Joanne Verheij, Michail Doukas, Patrick C.N. Rensen, Adriaan G. Holleboom, Maarten E. Tushuizen, Martin Giera

**Affiliations:** 1 Center for Proteomics and Metabolomics, Leiden University Medical Center, Leiden, The Netherlands; 2 Department of Pharmaceutical Chemistry, Faculty of Pharmacy, Universitas Gadjah Mada, Yogyakarta, Indonesia; 3 Division of Experimental Vascular Medicine, Department of Internal Medicine, Amsterdam UMC, Amsterdam, The Netherlands; 4 Department of Pathology, Amsterdam UMC, Amsterdam, The Netherlands; 5 Department of Pathology, Erasmus MC, Rotterdam, The Netherlands; 6 Division of Endocrinology, Department of Medicine, and Einthoven Laboratory for Experimental Vascular Medicine, Leiden University Medical Center, Leiden, The Netherlands; 8 Department of Gastroenterology and Hepatology, Leiden University Medical Center, Leiden, The Netherlands; 7 Division of Vascular Medicine, Department of Internal Medicine, Amsterdam UMC, Amsterdam, The Netherlands

**Keywords:** cholesterol, lipids, lipotoxicity, liver, phospholipids/phosphatidylcholine, MASL, MASH, biomarker, NITs

## Abstract

The accumulation of cholesterol and other lipids leading to hepatic lipotoxicity drives the progression of metabolic dysfunction-associated steatotic liver (MASL) to metabolic dysfunction-associated steatohepatitis (MASH), the advanced progressive stage of metabolic dysfunction-associated steatotic liver disease (MASLD). For MASH diagnosis, liver biopsy remains the reference standard, despite its invasiveness and limitations. Thus, this study aimed to find blood-derived lipid markers for MASH. We investigated serum samples from 86 patients with histologically characterized MASLD, spanning the disease spectrum (i.e. 62 patients with MASL (Fibrosis grade 0–4) and 24 patients with MASH (Fibrosis grade 2–4) with a balanced distribution of hepatocellular carcinoma) and analyzed sterol composition and lipidome. To identify the presence of MASH, logistic regression was performed on each candidate either in a single or combination with various clinical parameters. Serum levels of desmosterol and phosphatidylcholine are increased in patients with MASH compared to those with MASL. After exclusion of patients using lipid lowering drugs, an increase was also found in serum levels of cholesterol, cholesterol ester, lysophosphatidylcholine, lysophosphatidylethanolamine, phosphatidylethanolamine, and several individual lipid species. The ROC curve of each lipid candidate show the potential use of desmosterol, phosphatidylcholine, and a panel of lipid species in combination with alanine aminotransferase as potential diagnostic markers, characterized by a respective AUROC of 0.79 (95% CI 0.66–0.92), 0.80 (95% CI 0.64–0.97), and 0.91 (95% CI 0.82–1.00). Serum sterol and lipidome markers are characterized by strong AUROC results to distinguish with high accuracy MASH from MASL, potentially paving the way for future MASH biomarker development.

## Graphical Abstract

**Figure:**
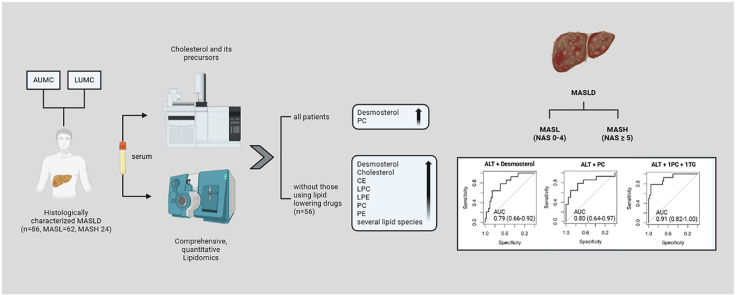

Metabolic dysfunction-associated steatotic liver disease (MASLD) is defined as excessive lipid droplet accumulation in hepatocytes in combination with at least one cardiometabolic risk factor, such as obesity, type 2 diabetes mellitus (T2DM), hypertension, or dyslipidemia, in the absence of other causes of liver disease (1). Abdominal, particularly visceral, obesity is strongly associated with MASLD, caused by a high flux of free fatty acids from insulin-resistant adipocytes to the liver in combination with increased hepatic lipogenesis driven by hyperinsulinemia (2, 3). MASLD affects 30% of the global adult population (4) with markedly higher prevalence in specific groups, such as individuals with overweight and obesity (5), and patients with T2DM (6). Also, due to the growing numbers of these populations, MASLD has become a major contributor to overall chronic liver disease (4).

MASLD with only isolated steatosis, referred to as metabolic dysfunction-associated steatotic liver (MASL), can progress to its progressive stage, metabolic dysfunction-associated steatohepatitis (MASH) characterized by necroinflammation and ballooning of hepatocytes (7, 8), and the induction of an ultimately maladaptive damage and repair response, leading to excessive extracellular matrix (ECM) deposition termed fibrosis (9, 10). Why only a subset of patients with MASLD progresses to the advanced stage of steatohepatitis remains poorly understood; however, differences in body weight, lipid and glucose metabolism, gut microbiome composition, and immune system may contribute to this process (11). Patients with MASH are at higher risk of progression to incremental stages of fibrosis, and subsequently advancement to cirrhosis and/or hepatocellular carcinoma (HCC), significant drivers of liver-related mortality (12, 13). This underscores the importance of detecting steatohepatitis when assessing MASLD severity, yet this has proven intensely difficult, in stark contrast to non-invasive tests (NITs) for steatosis and fibrosis (14). Interventions to treat or prevent MASH include lifestyle and dietary interventions, participation in clinical trials, and bariatric surgery. Only last year, the selective thyroid hormone receptor beta (THRβ)-selective agonist resmetirom was conditionally approved by the FDA as the first drug for the treatment of fibrotic MASH (15, 16).

At present, the gold standard for MASH staging is liver biopsy. However, this procedure poses significant risks, such as liver bleeding, sampling error, and high intra- and inter-observer variability, jointly limiting the use of biopsies in clinical routine (14, 17). Liver imaging by magnetic resonance imaging (MRI) or FibroScan, a more frequently used test, can only identify the presence of steatosis and fibrosis but not key features of MASH such as inflammation and ballooning (8, 18). Therefore, NITs for MASH are urgently needed.

Extensive studies for finding potential NITs for MASH or fibrotic MASH have been conducted. Many candidates, such as various lipid species (18, 19), proteins (20, 21), microRNA (21), or imaging (22, 23), have been proposed. However, none of them has as yet been approved for the diagnosis of MASH (24). Simonen *et al.* described increased levels of serum desmosterol in patients with MASH compared to patients with simple steatosis and a strong correlation between serum desmosterol levels and liver cholesterol levels (25). These findings are in line with studies reporting excessive amounts of free cholesterol (FC) and cholesterol crystals in MASH but not liver with simple steatosis (26, 27). Moreover, excess fatty acids, diglycerides (DG), and ceramides (CER) are known to be toxic to the liver and may contribute to disease development (28, 29). We consequently reasoned that a lipidome-wide analysis in combination with sterol analysis might further advance our understanding of pathophysiology and potentially result in advanced diagnostic markers for MASH.

In this study, we aimed to investigate and validate the potential use of desmosterol as a candidate biomarker for MASH. In addition, given the fact that lipids are main drivers of MASL pathophysiology and desmosterol has been described to activate the liver X receptor (LXR), a master regulator of lipid metabolism (30), we next questioned whether other lipid classes and/or species could serve as additional MASH markers in our cohort. We applied gas chromatography-mass spectrometry (GC-MS) (31) and quantitative differential mobility spectrometry-shotgun lipidomics (DMS-SLA) (32) to analyze serum samples from 86 patients spanning across several stages of MASL and MASH.

## Materials and Methods

### Study population

The study population consisted of patients originating from two academic centers, the Amsterdam MASLD-MASH cohort ANCHOR from Amsterdam University Medical Center (AUMC) and a cohort from Leiden University Medical Center (LUMC). Patients were included if they were ≥ 18 years of age, had liver steatosis detected by imaging with the presence of at least one cardiometabolic risk factor, and no known other cause of liver disease. Patients with excessive alcohol consumption (women ≥20 g/day, men ≥30 g/day), being pregnant, or receiving any specific drug treatment (e.g., methotrexate, anticoagulants) were excluded from the study. The study was approved by the ethics committee of AUMC (registered in the Dutch Trial Register NTR7191) and the ethics committee of LUMC (B21.045). Written informed consent was obtained from all patients, and the study was conducted in accordance with the Declaration of Helsinki.

### Study design

**Fig. 1:**
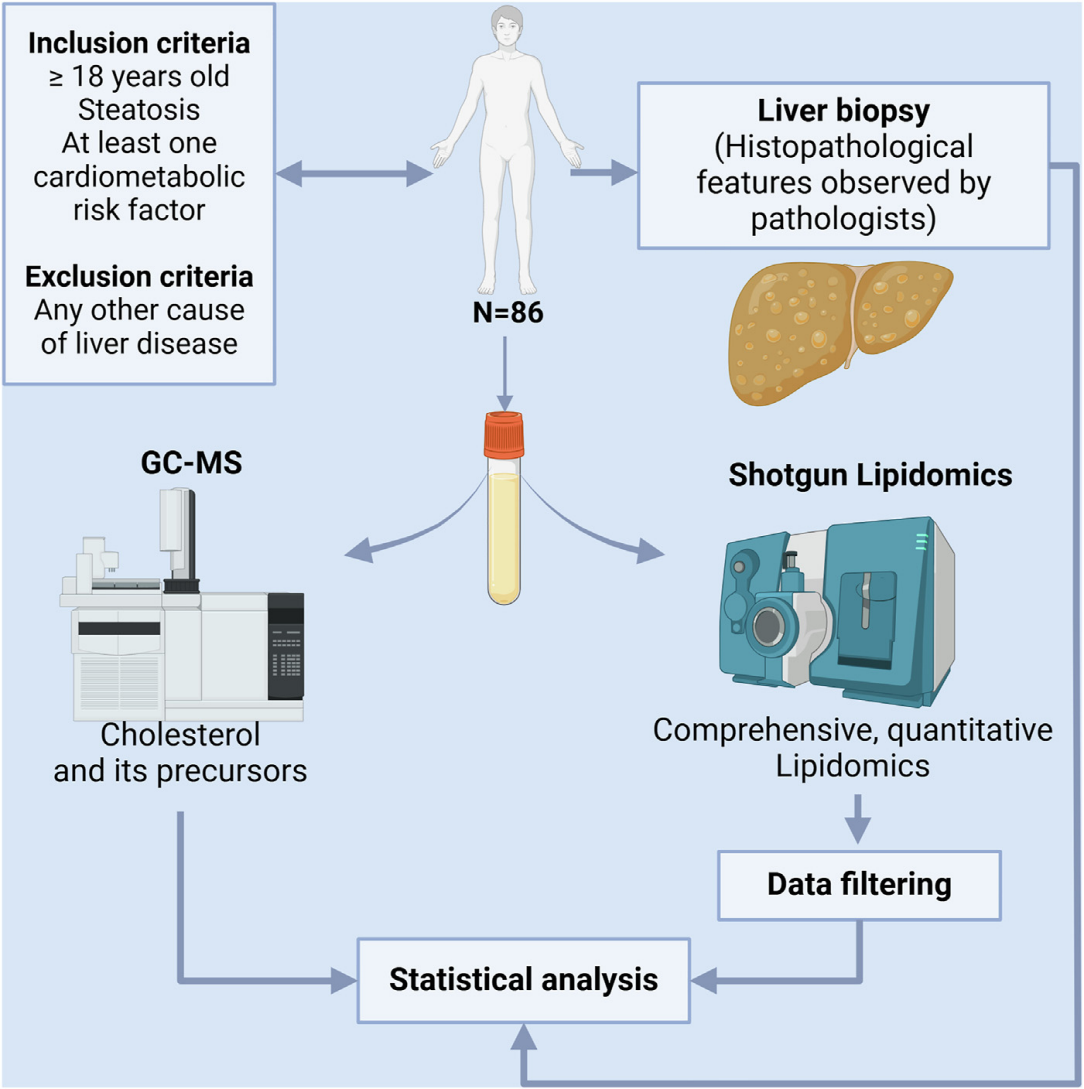
Overall study design.

After an overnight fast, blood samples were drawn on the same day as ultrasound-guided liver biopsy. Blood was collected, allowed to clot at room temperature for ≥ 30 min, and serum was obtained after centrifuging at 1,550 g for 20 min and stored at −80°C before being analyzed. Body weight and height were measured. Metabolic parameters, including fasting glucose, HbA1c, triglyceride, HDL-cholesterol, and the liver enzymes aspartate aminotransferase (AST) and alanine aminotransferase (ALT) were obtained from clinical chemistry analysis according to standard procedures. The overall study scheme is shown in Figure 1.

### Liver biopsy and liver histology

Liver biopsies were taken by trained radiologists in both academic centers following the local standard procedure. Histological feature assessment of all liver biopsies was performed by two pathologists (J.V. and M.D.), resulting in tandem scoring minimizing inter-observer variability. The assessments were done based on standard criteria for steatosis, lobular inflammation, ballooning, and fibrosis score. To specifically compare MASL and MASH, we divided the patients by their NAFLD Activity Score (NAS), being the composite score of steatosis, lobular inflammation, and ballooning. As defined by the Nonalcoholic Steatohepatitis Clinical Research Network (NASH CRN) a NAS of ≥ 5 reflects a diagnosis of MASH; thus, we grouped the patients with NAS of ≥ 5 into the MASH group, and the patients with NAS of 0–4 into the MASL group regardless of their fibrosis score (33). To assess correlations between lipidomic profile and fibrosis severity, patients were divided into four groups, in which fibrosis groups 1, 2, 3, and 4 equal fibrosis scores 0–1 (1a, 1b, and 1c), 2, 3, and 4, respectively.

### Sterol quantification

Total cholesterol and precursors were quantified by GC-MS analysis using an Agilent VF-5 ms (30 m × 0.25 mm i.d. × 0.25 μm) column and helium as the carrier gas at a constant flow rate of 1 ml/min following published protocols (31). Briefly, 10 μl of serum sample, internal standards (IS; containing cholestane, cholesterol d7, and desmosterol d6), ethanol, and sodium hydroxide solution (10 M) were combined in a glass vial (final concentration of sodium hydroxide was 1 M in 80% v/v of ethanol), flushed with nitrogen, capped, vortexed, and incubated at 70°C for 1 h. After cooling, the solution was purified by solid phase extraction (SPE) using Phenomenex Strata™-X 33 μm Polymeric Reverse Phase SPE cartridges. Briefly, SPE columns were activated using 200 μl of methanol and water, followed by sample loading. Columns were then washed with 500 μl of water and sterols eluted using 300 μl of methyl formate. After drying under a gentle stream of nitrogen, sterols were derivatized for 30 min at room temperature using a mixture of N-methyl-N-(trimethylsilyl)trifluoroacetamide, trimethylchlorosilane, and N-(trimethylsilyl)imidazole, and analyzed by GC-MS in single ion-monitoring mode. A detailed description of the preparation procedure and GC-MS system settings can be found elsewhere (31). Each sterol concentration is expressed in μM.

### Shotgun lipidomics analysis

Shotgun lipidomics analysis across 17 different lipid classes was accomplished by differential mobility spectroscopy coupled with tandem mass spectrometry operated with the shotgun lipidomics assistant software (DMS-SLA platform) (32, 34, 35). An amount of 25 μl of serum was spiked with IS, covering 74 deuterated lipid species (UltimateSPLASH™ ONE in combination with oleic acid-d9 (dFA 18:1), C13-dihydroceramide-d7 (d18:0-d7/13:0), C15-glucosyl(β)ceramide-d7 (d18:1-d7/15:0), C15-lactosyl(β)ceramide-d7 (d18:1-d7/15:0), and 15:0-18:1-d7-PA) across 17 different lipid classes (with known concentration for each of them). Lipids were subsequently extracted using a methyl tert-butyl ether-based protocol. Each lipid species was quantified by the SLA software based on its average intensity divided by the average intensity of the most structurally similar IS multiplied by its corresponding concentration. A detailed preparation procedure and the DMS-SLA setting can be found elsewhere (32, 34, 35). Similarly, water blanks and plasma quality controls (QC) were prepared using either LC-MS grade water or human citrate plasma (Sigma P9523). Initial data filtering was based on QC plasma analysis. All lipid species with a relative standard deviation (RSD) of their respective concentration > 30% were removed. Subsequently, only lipid species fulfilling the following criteria were included for analysis: 1) having a signal of > 2 times the blank, 2) being present in > 80% of all samples, and 3) being re-included if 2) does not apply, but present in ≥ 80% of the samples of a study group being tested. The so obtained lipidomics data consisting of roughly 730 individual lipid species (across 16 different lipid classes with dihydroceramides and ceramides are combined together as ceramides) was analyzed using the multi-omics analysis software iSODA (1.0.16) (36). For details about the lipid species annotation level of the platform please refer to reference (32). Moreover, for all lipid species, the LipidMaps (37) annotation system has been adopted, for triglyceride (TG) species, the platform is capable of detecting the total carbon and double bond number for example indicated as follows, TG 52:3. Moreover, one of the three fatty acid (FA) side chains with its carbon and double bond number can be identified, being indicated as TG 52:3-FA18:1.

### Tandem mass spectrometric analysis of candidate lipids

To verify the identity of PC 16:0_16:1 and TG 60:12-FA22:6 we collected tandem MS spectra (MS/MS) from a pooled MASLD sample on a reversed phase liquid chromatography high-resolution mass spectrometry platform (LC-MS/MS). Briefly, the sample was extracted using a methyl tert-butyl ether-based protocol. The extracted sample was analyzed using an LC-MS/MS based method. A Sciex ExionLC AD was used to deliver a gradient of water:acetonitril 80:20 (eluent A) and water:acetonitril:2-propanol 1:9:90 (eluent B). Both eluents contained 5 mM ammonium formate and 0.05% formic acid. The applied gradient, with a column flow of 300 μl/min, was as follows: 0 min 40% B, 10 min 100% B, 12 min 100% B. A Phenomenex Kinetex C18, 2.7 μm particles, 50 × 2.1 mm was used as column. The injection volume was 10 μl. The column oven was set to 50°C. The MS was a Sciex ZenoTOF 7,600+ operated in positive (ESI+) and negative (ESI-) ESI mode, with the following conditions: Ion Source Gas 1, 2, and Curtain gas 30 psi, temperature 350°C, acquisition range m/z 100–1,200, IonSpray Voltage 5500 V (ESI+) and −4500 V (ESI-), declustering potential 80 V (ESI+) and −80 V (ESI-). An information dependent acquisition (IDA) method in ESI- mode was used to confirm the identity of PC 16:0_16:1, with the following conditions for MS analysis: collision energy −10, acquisition time 250 ms and for MS/MS analysis: collision energy −45, collision energy spread 15, acquisition time 40 ms, time bins to sum 8, zeno pulsing on, zeno threshold 20,000 cps. The IDA switching criteria were set as: for ions greater than m/z 300, which exceed 200 cps, exclude former target for 2 s, exclude isotopes within 1.5 Da, max. candidate ions 20. To confirm the identity of TG 60:12_FA22:6 a targeted MS/MS method was used, with the following conditions for MS analysis: collision energy 10, acquisition time 250 ms and for MS/MS analysis: precursor ion 968.77 Da, collision energy 45, collision energy spread 15, acquisition time 250 ms, time bins to sum 8, zeno pulsing on, zeno threshold 20,000 cps. The acquired MS/MS spectra were manually annotated.

### Statistical analysis

Chi-square or Fisher's exact test was used for comparing categorical data, while non-parametric Wilcoxon and Kruskal-Wallis tests were used for comparing continuous data. In case the result of the Kruskal-Wallis test was significant (*P* value < 0.05), Dunn’s post hoc test with Benjamini-Hochberg multiple testing correction was applied. Receiver operating characteristic (ROC) curve analysis was performed to assess the discriminative ability of the candidate markers. Analyses were conducted using SPSS 29 and R (4.4.2) with tidyverse (2.0.0), ggpubr (0.6.0), rstatix (0.7.2), corrplot (0.95), caret (7.0-1), ggcorrplot (0.1.4.1), tidyr (1.3.1), ggplot2 (3.5.1), ggrepel (0.9.6), and pROC (1.18.5) packages.

## Results

### Characteristics of the study population

A total of 86 biopsy proven patients with MASLD were enrolled at AUMC (n = 52) and LUMC (n = 34) (Fig. 1). To compare the serum lipid profiles between patients with MASL and MASH we grouped them based on their histological features, resulting in 62 patients in the MASL group and 24 patients in the MASH group. The clinical characteristics of the study population are summarized in Table 1. Importantly, no statistical differences in sex distribution were found between the two groups. Also, no differences were observed in age, BMI, the levels of fasting glucose, HbA1c, triglyceride, and HDL-cholesterol. In contrast, the AST and ALT levels were significantly higher in MASH compared to MASL (61 vs. 40 U/L and 70 vs. 54 U/L, respectively). In terms of histopathological features, differences were observed in the distribution of steatosis, lobular inflammation, ballooning, and fibrosis score, yet HCC distribution did not differ.

**Table 1:** Clinical characteristics of the patients by liver phenotype

Parameter	MASL (n = 62)	MASH (n = 24)	*P*-value
Sex (M)	41 (66.1%)	14 (58.3%)	0.499
Age (years)	52.0 (43.8–62.3)	50.5 (38.3–62.0)	0.722
BMI (kg/m^2^)	31.6 (29.0–35.5)	32.5 (29.9–36.2)	0.637
Fasting glucose (mmol/L)	6.2 (5.5–8.7)	6.6 (5.6–7.3)	0.684
	NA = 2	NA = 1	
HbA1c (mmol/mol)	40 (36–53)	41 (38–51)	0.741
	NA = 16	NA = 8	
Triglyceride (mmol/L)	1.4 (1.0–2.3)	1.9 (1.2–2.2)	0.356
	NA = 7	NA = 3	
HDL-cholesterol (mmol/L)	1.1 (1.0–1.4)	1.2 (1.0–1.6)	0.237
	NA = 5	NA = 3	
AST (U/L)	40 (34–53)	61 (38–102)	0.007
ALT (U/L)	54 (39–72)	70 (46–142)	0.038
Steatosis			<0.001ᶧ
0	6 (9.7%)	0 (0%)	
1	31 (50.0%)	2 (8.3%)	
2	22 (35.5%)	5 (20.8%)	
3	3 (4.8%)	17 (70.8%)	
Lobular inflammation			0.007ᶧ
0	8 (12.9%)	0 (0%)	
1	50 (80.6%)	17 (70.8%)	
2	4 (6.5%)	7 (29.2%)	
Hepatocyte ballooning			<0.001
0	29 (46.8%)	0 (0%)	
1	28 (45.2%)	11 (45.8%)	
2	5 (8.1%)	13 (54.2%)	
NASH CRN fibrosis grade			0.01ᶧ
0-1 (1a, 1b, 1c)	16 (25.8%)	0 (0%)	
2	23 (37.1%)	8 (33.3%)	
3	12 (19.4%)	9 (37.5%)	
4	11 (17.7%)	7 (29.2%)	
HCC (no/yes/NA)	49 (79.0%)/13 (21.0%)/0 (0%)	21 (87.5%)/3 (12.5%)/0 (0%)	0.539ᶧ

Data are shown as frequency (%) (categorical data) and median (IQR) (numerical data). Statistical tests performed were Chi-square or Fisher exact test (ᶧ) (categorical data) and Wilcoxon (numerical data).

### Serum desmosterol levels are higher in MASH compared to MASL

**Fig. 2:**
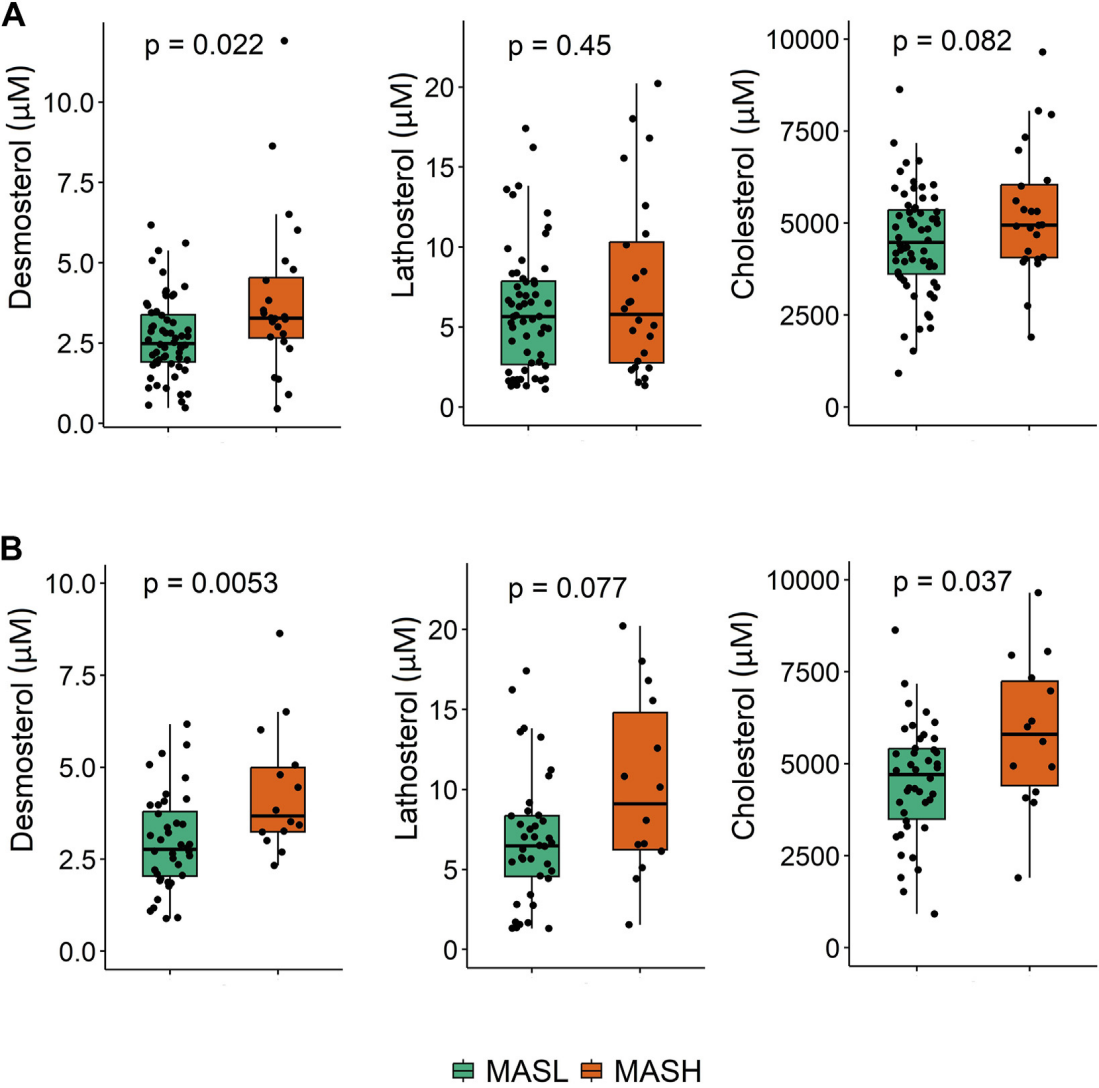
Serum desmosterol, lathosterol, and cholesterol in patients with MASL and MASH, including those using lipid-lowering drugs (n = 86) (A) and excluding those using lipid-lowering drugs (n = 56) (B). Statistical analysis was performed by the Wilcoxon test.

As shown in Figure 2, we found that serum desmosterol levels are higher in patients with MASH compared to patients with MASL (3.3 vs. 2.5 μM; *P* = 0.022), while lathosterol (*P* = 0.450) and cholesterol (*P* = 0.082) did not differ (Fig. 2A). Given the fact that lipid-lowering drugs (i.e., statins, fibrates, ezetimibe, PCSK9 inhibitors, or a combination thereof) potentially affect cholesterol biosynthesis rates, we next excluded lipid-lowering drugs users from our analysis. This led to a significantly stronger difference in desmosterol between patients with MASH and MASL (3.7 vs. 2.8 μM; *P* = 0.0053), and to an additional significant difference for cholesterol (5,801 vs. 4,707 μM; *P* = 0.037).

### Profiles of lipid classes in the serum of patients with MASH compared to MASL

**Fig. 3:**
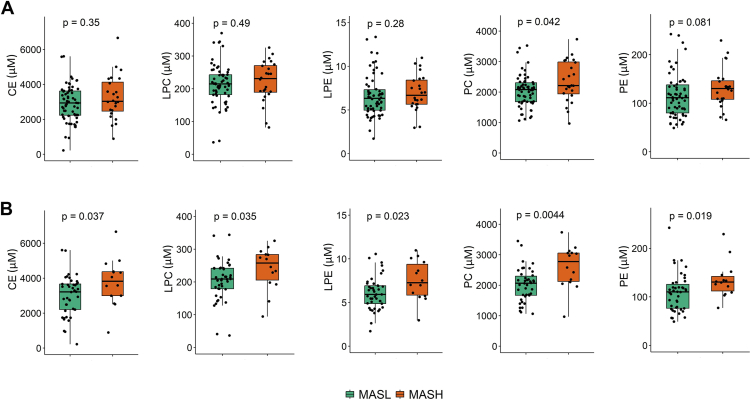
Serum levels of CE, LPC, LPE, PC, and PE between patients with MASL and MASH, including those using lipid-lowering drugs (n = 86) (A), and excluding those using lipid-lowering drugs (n = 56) (B). Statistical analysis was performed by the Wilcoxon test.

Using our DMS-SLA platform we next quantified more than 800 lipid species across 17 lipid classes including cholesteryl ester (CE), dihydroceramide (Cer d18:0), ceramide (Cer d18:1), diglyceride (DG), free fatty acid (FA), hexosylceramide (HexCER), lysophosphatidylcholine (LPC), lysophosphatidylethanolamine (LPE), lactosylceramide (LacCER), phosphatidic acid (PA), phosphatidylcholine (PC), phosphatidylethanolamine (PE), phosphatidylglycerol (PG), phosphatidylinositol (PI), phosphatidylserine (PS), sphingomyelin (SM), and triglyceride (TG). After data pre-processing, approximately 700 lipid species across 13 lipid classes remained for data analysis. The results are shown in Figure 3 as well as supplemental Figs. S1 and S2.

When analyzing the entire cohort, PC levels were higher in patients with MASH compared to MASL (2,212 vs. 2,081 μM; *P* = 0.042; Fig. 3A), while levels of CE, LPC, LPE, and PE did not differ significantly. After exclusion of users of lipid-lowering drugs, the difference in PC levels enlarged (2,777 vs. 2,067 μM; *P* = 0.0044), and levels of CE (3,825 vs. 3,213 μM; *P* = 0.037), LPC (258 vs. 209 μM; *P* = 0.035), LPE (7.3 vs. 5.9 μM; *P* = 0.023), and PE (131 vs. 110 μM; *P* = 0.019) were significantly higher in patients with MASH compared to MASL (Fig. 3B). Since predominantly phospholipids seemed to distinguish MASL from MASH, we combined all phospholipid classes (i.e., LPC, LPE, PC, PE, PI, and SM) but observed a significant difference only when removing lipid-lowering drugs users (3,753 vs. 2,978 μM; *P* = 0.0082) (supplemental Fig. S2).

Analysis at the lipid species level was accomplished by creating volcano plots. In general, the level of each lipid species was higher in patients with MASH compared to MASL. However, after Benjamini-Hochberg multiple testing correction, statistical significance was lost for all lipid species. Exclusion of lipid-lowering drug users showed significantly higher levels of CE16:1, some PC species (16:0_16:1, 16:0_20:5, 18:0_20:2, 18:0_20:5), and some TG species (54:7-FA16:1, 54:7-FA22:6, 58:6-FA16:0, 60:12-FA22:6) in patients with MASH compared to MASL (supplemental Figs. S3 and S4). Moreover, we did not detect significant changes in the FA side chains of the various lipid classes (i.e., double bond number and chain length) between groups (supplemental Figs. S5 and S6), without and with exclusion of lipid-lowering drug users.

### Cirrhosis is characterized by lower serum lathosterol and cholesterol levels

**Fig. 4:**
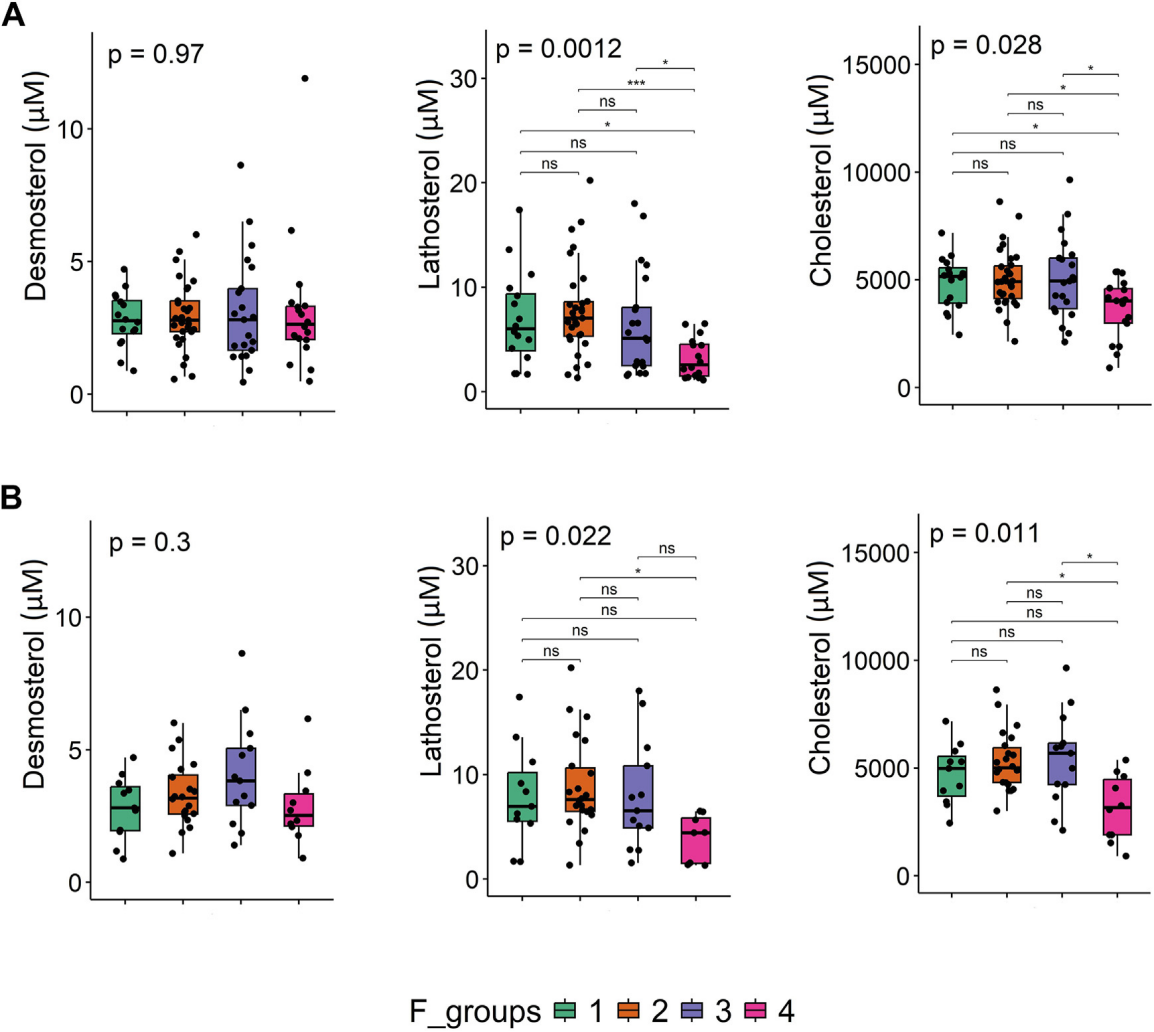
Sterol levels between patients with fibrosis, including those using lipid-lowering drugs (n = 86) (A), and excluding those using lipid-lowering drugs (n = 56) (B). Fibrosis group 1 (F score 0–1), group 2 (F score 2), group 3 (F score 3), and group 4 (F score 4). Statistical analysis was done by Kruskal-Wallis, and in cases where the *P*-value < 0.05, Dunn’s post hoc test was conducted, followed by Benjamini-Hochberg multiple testing correction. ∗*P* < 0.05, ∗∗∗*P* < 0.001 between two groups.

To investigate whether our lipid data might reveal markers for advanced fibrosis and cirrhosis, we grouped our patients according to fibrosis grade and compared their lipid profiles (Fig. 4, supplemental Figs. S7 and S8).

Serum levels of sterols did not differ between the various fibrosis scores (0–3), but lower levels of lathosterol and cholesterol were observed in cirrhosis (F score 4), without and with exclusion of those patients using lipid-lowering drugs (Fig. 4). Similar patterns were found across 8 different lipid classes (i.e., CE, Cer, DG, PC, PE, PI, SM, and TG), where lower lipid levels were only apparent in patients with cirrhosis. Level of lipids in other classes (i.e., FA, HexCER, LPC, LPE, and LacCER) remained stable across different fibrosis groups (supplemental Figs. S7 and S8).

### Exploring the potential of lipids as a candidate biomarker in MASH

**Fig. 5:**
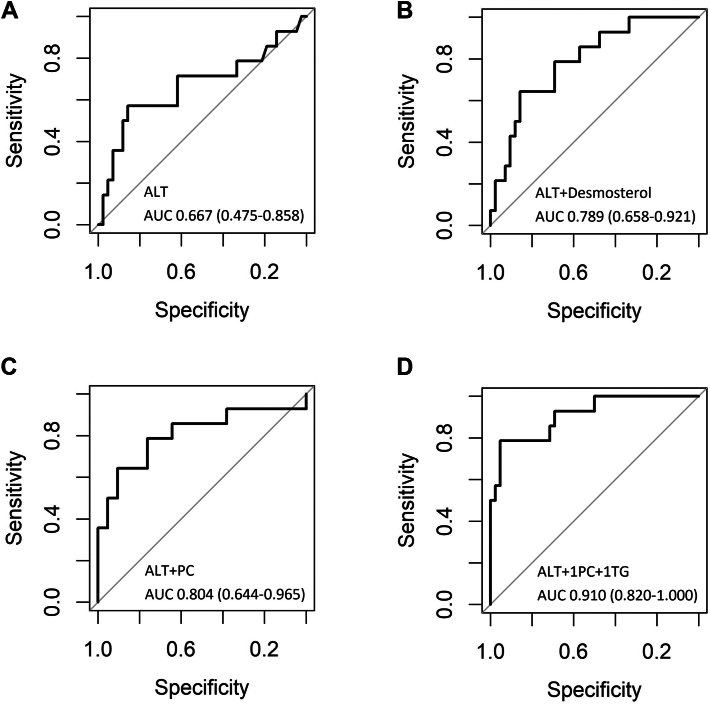
ROC curves of ALT (A) and a combination of ALT with desmosterol (B), PC (C), and a panel of lipid species (D).

Next, we performed logistic regression analysis for each of the statistically significant lipid features that allowed to differentiate MASH from MASL. Moreover, we built receiver–operating characteristic (ROC) curves to gain an overview on their discriminative power in predicting the presence of MASH in patients with MASLD. The areas under the ROC curves (AUROCs) of each lipid candidate as a single predictor were between 0.7 and 0.8 with no intersection with 0.5 for their 95% confidence intervals (CIs). In fact, they are superior over ALT, a measure of liver injury for several decades (24) (Fig. 5 and supplemental Table S1). We continued the investigation by combining lipid candidates with several clinical covariates and applied backward elimination. Inclusion of age, sex, BMI, the presence of T2DM, and ALT revealed that only ALT significantly contributed to the models and/or yielded the greatest incremental gain in AUROC. Therefore, we built ROC curves of each lipid candidate in combination with ALT, which increased AUROCs for most lipid candidates including desmosterol, PC, phospholipid, and several lipid species (AUROCs ≥ 0.8; Figure 5 and supplemental Table S1). Although the initial AUROCs were acceptable, we investigated whether combining lipids could enhance the performance. By combining lipids with low correlation and reintroducing ALT, we found that combination of ALT, PC 16:0_16:1, and TG 60:12_FA 22:6 yielding the highest AUROC (0.910) (Fig. 5, supplemental Table S2, and supplemental Fig. S9). The identity of both candidate lipids was confirmed by LC-MS/MS analysis (supplemental Fig. S10). To validate the models a cross validation (100x) was done. Each time the data was split into a training set (75%) and a test set (25%). The split was stratified to ensure that the proportion of MASH cases is equal between the training and test set. The results shows that the average of the AUROCs are in between 0.7 and 0.9 for all of the lipid candidates either as a single predictor or in combination (supplemental Tables S1 and S2).

## Discussion

Excess lipid accumulation in the liver of patients with MASLD induces lipotoxicity, ultimately promoting the development of MASH. An imbalance between lipolysis, lipid uptake, lipogenesis, lipid oxidation, and secretion contribute to lipid accumulation and changes liver lipid distribution (38). Our study in a cohort of patients with biopsy-proven MASLD confirms that serum levels of desmosterol, the penultimate precursor of cholesterol (30, 39, 40), is higher in MASH compared to MASL. In addition, we demonstrate that desmosterol, PC, as well as a panel of lipid species including PC 16:0_16:1 and TG 60:12_FA22:6, are highly accurate in distinguishing MASH from MASL, which may offer a promising NIT for disease diagnosis and monitoring disease progression.

The higher serum desmosterol levels in patients with MASH compared to MASL were previously hypothesized to be associated with an increased rate of cholesterol biosynthesis (25). Alternatively, increased levels of desmosterol (e.g., in macrophage foam cells) have been shown to result from feedback inhibition as a consequence of cholesterol overload (30). Excess cholesterol profoundly represses DHCR24, the enzyme responsible for the conversion of desmosterol to cholesterol in the Bloch pathway (30, 39, 40). Accumulation of desmosterol activates LXRs to enhance cholesterol efflux through upregulation of the cholesterol transporters (i.e., ABCA1 and ABCG1) and to reduce cholesterol influx through degradation of the LDL receptor, as well as suppresses genes involved in lipogenesis and the inflammatory response (30). Along these lines, it was recently found that inhibition of DHCR24 elevates desmosterol levels, thereby improving hepatic steatosis and inflammation (41). These results suggest a similar mechanism potentially being activated in Kupffer cells. Since desmosterol is secreted from the liver into the circulation within very low-density lipoproteins (VLDL) and/or high-density lipoproteins (HDL), serum levels of desmosterol likely correlate with desmosterol levels in the liver (25, 40, 41). The recently FDA conditionally approved drug resmetirom is hypothesized to increase β oxidation of fatty acids and restoration of normal mitochondrial function (resulting in a reduction of the liver fat content) and reduce VLDL production and secretion (resulting in a reduction of plasma TG, LDL-cholesterol, and apolipoprotein B) which may convey protection from atherosclerotic cardiovascular disease (15, 42). Hence, patients with MASH receiving resmetirom may likely have diminished serum desmosterol concentrations.

With regard to lipidomic changes (supplemental Figs. S3 and S4), the serum levels of almost all lipid classes are higher in MASH compared to MASL, potentially indicating broadly elevated circulating lipids. Although FA, DG, and CER were previously reported to play a role in the pathophysiology of MASH (28, 29), in our cohort, we did not find increased serum levels in patients with MASH compared to MASL (supplemental Figs. S1 and S2). Instead, we found higher serum levels of free cholesterol (FC) (supplemental Fig. S11) estimated by subtracting the CE fraction from the total cholesterol analysis result. Besides generally elevated levels of lipid classes, several lipid species (i.e., CE 16:1, several PCs (16:0_16:1, 16:0_20:5, 18:0_20:2, 18:0_20:5) and TGs (54:7-FA16:1, 54:7-FA22:6, 58:6-FA16:0, 60:12-FA22:6)) were found as class specific markers significantly higher in MASH compared to MASL (supplemental Fig. S4). When further investigating correlations of lipid markers with histopathological features (i.e., steatosis, lobular inflammation, and ballooning) and their composite score (NAS) we found that almost all lipids correlated with steatosis and NAS, but only desmosterol and PC 16:0_16:1 correlated with both lobular inflammation (r = 0.237, *P* = 0.085 and r = 0.305, *P* = 0.022, respectively) and ballooning (r = 0.403, *P* = 0.002 and r = 0.514, <0.001, respectively) (supplemental Table S3). In terms of fibrosis, no lipid-derived markers differentiating fibrosis stages were observed, and only patients with cirrhosis presented with a significant decrease in lathosterol, cholesterol, CE, Cer, DG, PC, PE, PI, SM, and TG.

From the above data, we observed that the exclusion of patients using lipid-lowering drugs affects the analysis result of several lipids (i.e., desmosterol, cholesterol, CE, LPC, LPE, PC, PE, phospholipid, and lipid species) (Figs. 2 and 3, supplemental Figs. S1–S4). Lipid-lowering drugs act by inhibiting cholesterol synthesis or cholesterol absorption, reducing VLDL production, or increasing the expression of low-density lipoprotein receptor (LDL-R), thereby decreasing lipid secretion or increasing lipid uptake, and subsequently causing a decline in circulating lipids (43). As these mechanisms potentially influence levels of lipid markers under investigation, we consequently excluded patients using lipid-lowering drugs and compared the obtained results to the entire study cohort.

Finally, we explored whether the observed lipid candidate markers were able to discriminate patients with MASL and MASH. As presented here, particularly combinations of ALT with desmosterol, PC, and several selected lipid species produced acceptable accuracies (AUROC = 0.789–0.910). The so-generated lipid-based models outperform the performance of either ALT or AST (Fig. 5, supplementary Fig. S12, and supplemental Table S1). Previous studies reported cytokeratin 18 M30, cytokeratin 18 M65 (20), and a panel of TG (18) with AUROCs 0.75 (0.69–0.82), 0.82 (0.69–0.91), and 0.79 (0.75–0.83), respectively, for MASH diagnosis. In terms of fibrotic MASH, several candidates have been proposed, such as FAST (a combination of LSM, CAP, and AST) with AUROC 0.80 (0.76–0.85) (23), MAST (a combination of MRI-PDFF, MRE, and AST) with AUROC 0.93 (0.88–0.97) (22), NIS4 (a combination of miR-34a-5p, YKL-40, alpha2-macroglobulin, and HbA1c) with AUROC 0.815 (0.786–0.844) (24), and NIS2+™ (a combination of miR-34a-5p, YKL-40, sex, sex∗miR-34a-5p) with AUROC 0.813 (0.795–0.832) (21). In this regard, our here presented lipid derived biomarkers in combination with ALT show very promising results that warrant further exploration and validation. Compared with earlier reports, our candidate panel is more streamlined and potentially more readily translatable. This particularly counts for the pairing of ALT with desmosterol, the PC class, or an abundant PC species, as shown here for PC 16:0_16:1. For the latter, it has to be however noted that individual lipid-species markers still face practical hurdles that could restrict routine use (see study limitations). In turn, we propose that the streamlined ALT–desmosterol (±PC) panel be prioritized for prospective validation as a cost-effective, easily implementable screening tool for MASH in routine clinical practice.

## Conclusions

We show that MASH, compared to MASL, is characterized by higher levels of serum lipids (i.e., desmosterol, cholesterol, CE, LPC, LPE, PC, PE, and some lipid species) of which several show great potential for distinguishing patients with MASH from MASL, particularly when combined with ALT. Moreover, we confirm the potential use of desmosterol as marker of MASH, and provide several additional potential lipid markers (e.g., PC and a panel of lipid species (PC 16:0_16:1 and TG 60:12-FA22:6)). Our study paves the way for further development and validation of lipid markers in the diagnosis of MASH versus MASL within patients with MASLD.

## Study limitations

We recognize that rigorous validation is essential for demonstrating the reproducibility of our findings, yet a second patient cohort was not available to us. Nonetheless, in this study we provide evidence for the potential use of desmosterol as a marker for MASH, independently confirming the work of Simonen *et al.* (25).

In our lipidomic workflow we used a shotgun lipidomics platform with single-point IS calibration. For future applications, we recommend adopting multipoint calibration with labelled IS that span expected concentration ranges observed in human biospecimen. Because many reference standards are commercially unavailable, structural confirmation of key lipids will also be crucial. Here, we collected high-resolution LC–MS/MS spectra for the two lipids yielding the highest AUROC, PC 16:0_16:1 (ESI-) and TG 60:12-FA22:6 (ESI+), and verified their identities by manual spectral interpretation.

However, from a clinical translational perspective and given the analytical complexity of comprehensive lipid species identification and quantification, we propose that a streamlined panel combining serum ALT with desmosterol and either a representative phosphatidylcholine or total phosphatidylcholine measurement would be far more practical for routine clinical use.

## Data availability

The (raw) data will be made available upon reasonable request.

## Supplemental data

This article contains supplemental data.

## Conflict of interest

The authors declare the following financial interests/personal relationships which may be considered as potential competing interests: M. N. is founder and a scientific advisory board member of Caelus Pharmaceuticals and Advanced Microbiome Interventions, the Netherlands. However, none of these are relevant for the current paper. The other authors declare no competing of interests.
