# Supplementary material for: Non-invasive identification of steatohepatitis in patients with MASLD using a sterol and lipidomic signature
Source: J Lipid Res. 2025 Jun 20;66(8):100845. doi: 10.1016/j.jlr.2025.100845 (PMC12284782; doi:10.1016/j.jlr.2025.100845)
Supplement: Supplemental data [file mmc1.docx]

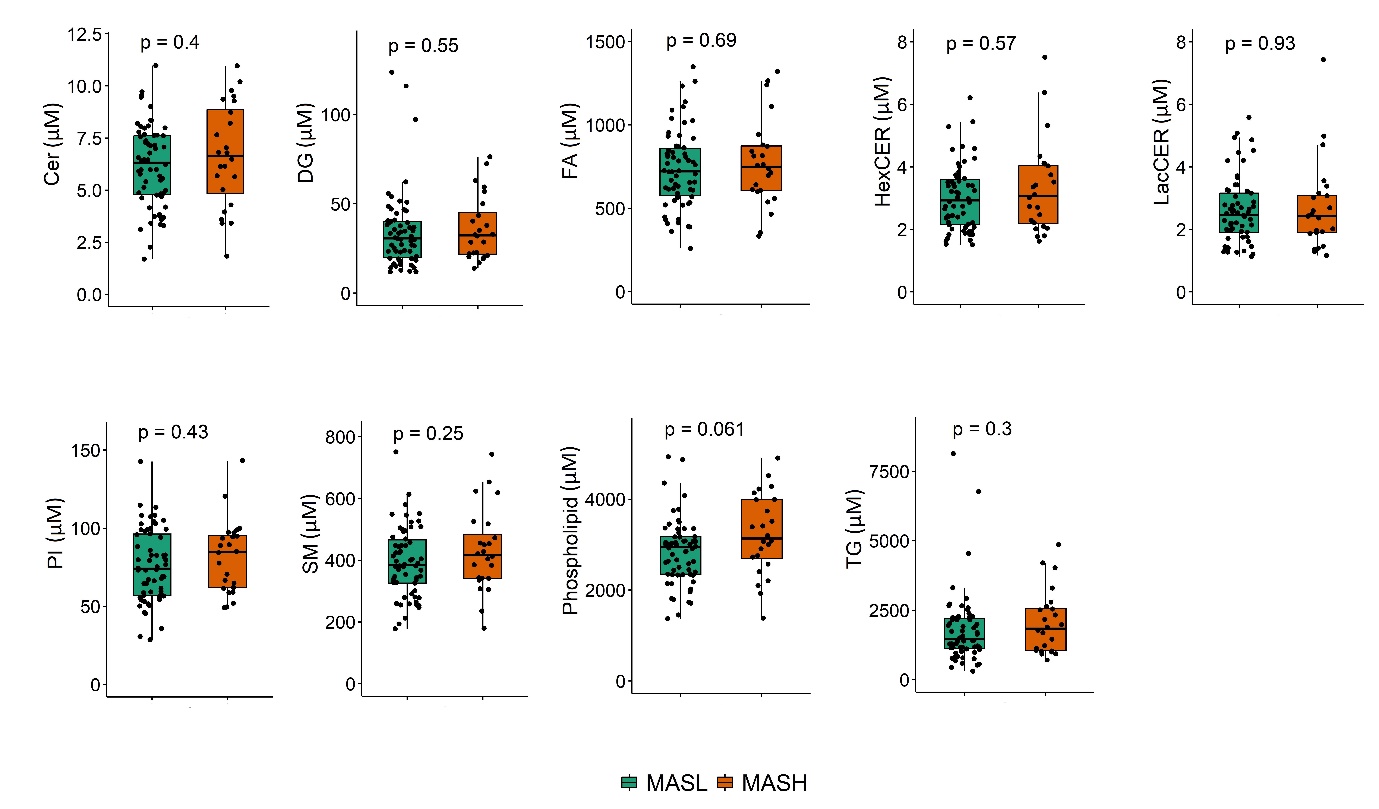


**Supplementary Figure 1.** Serum Cer, DG, FA, HexCER, LacCER, PI, SM, Phospholipid, and TG between patients with MASL and MASH, including those using lipid-lowering drugs (n=86). Statistical analysis was performed by Wilcoxon test.


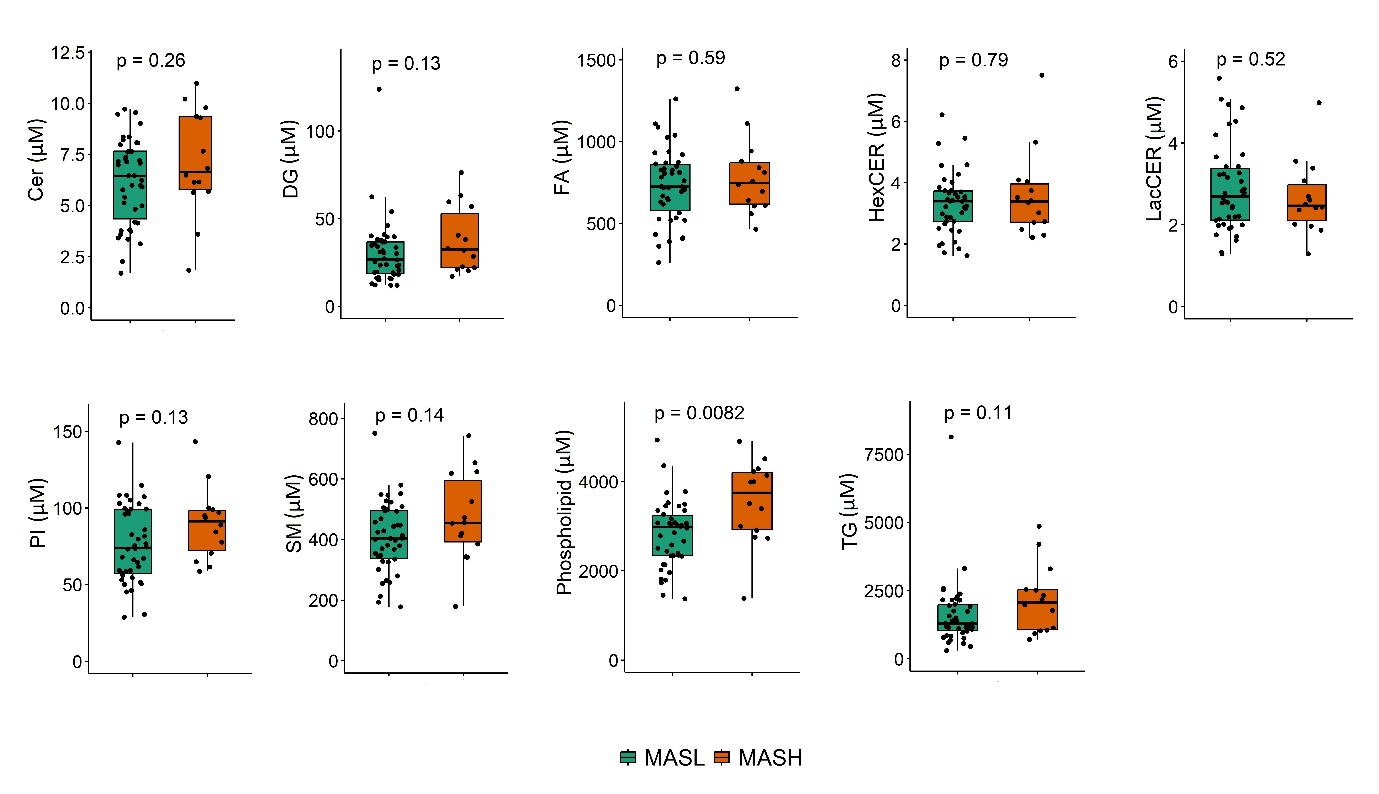


**Supplementary Figure 2.** Serum Cer, DG, FA, HexCER, LacCER, PI, SM, Phospholipid, and TG between patients with MASL and MASH, excluding those using lipid-lowering drugs (n=56). Statistical analysis was performed by Wilcoxon test.


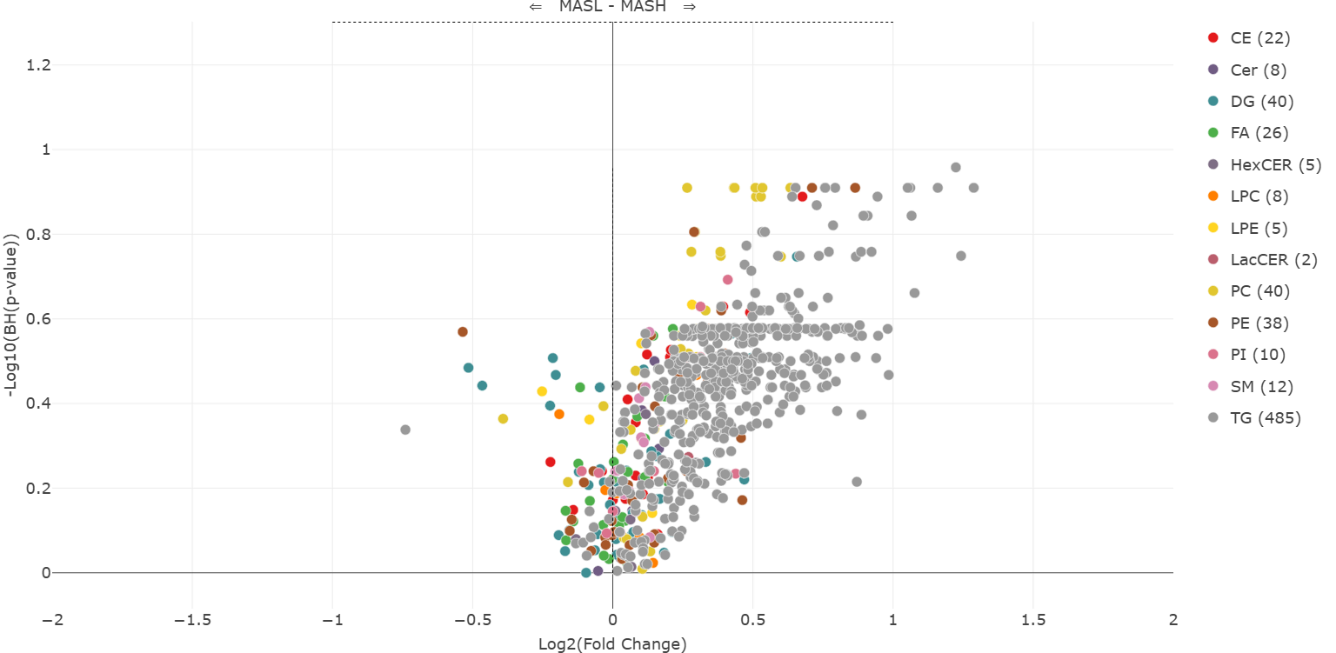


**Supplementary Figure 3.** Volcano plot of serum lipid species between patients with MASL and MASH, including those using lipid-lowering drugs (n=86). The analysis was performed using iSODA using Wilcoxon test followed by Benjamini-Hochberg multiple testing correction.


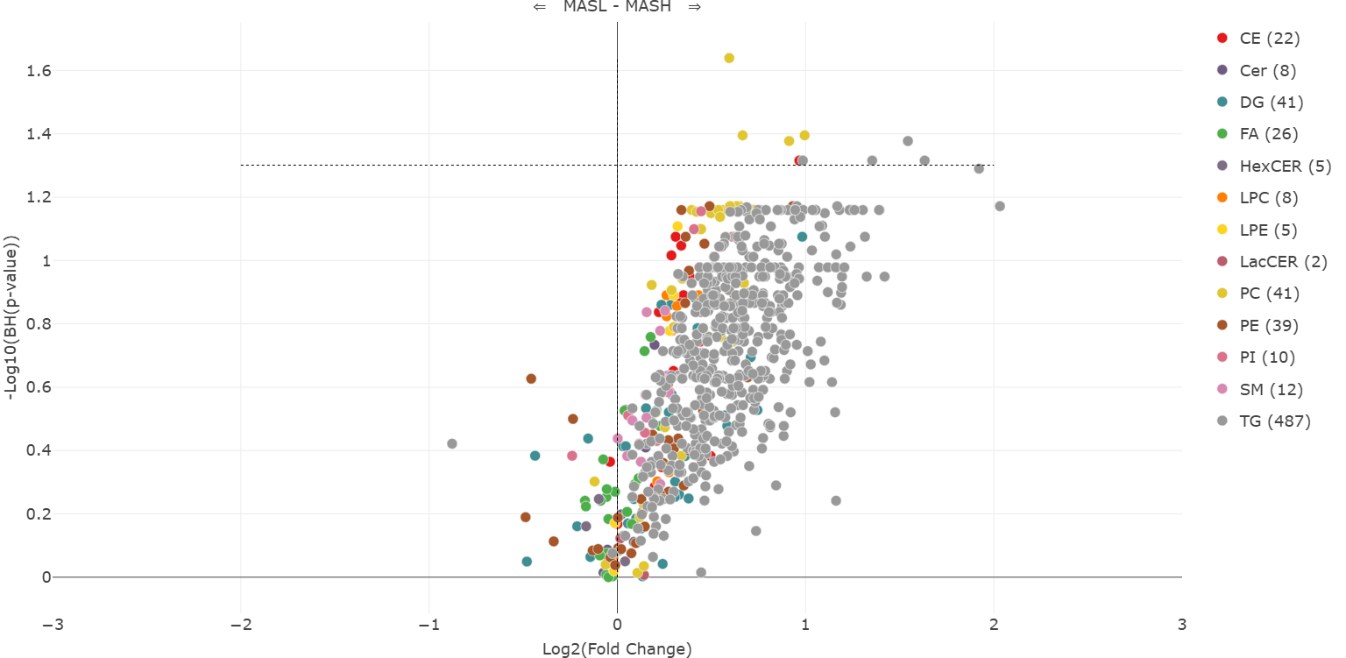


**Supplementary Figure 4.** Volcano plot of serum lipid species between patients with MASL and MASH, excluding those using lipid-lowering drugs (n=56). The analysis was performed using iSODA using Wilcoxon test followed by Benjamini-Hochberg multiple testing correction.


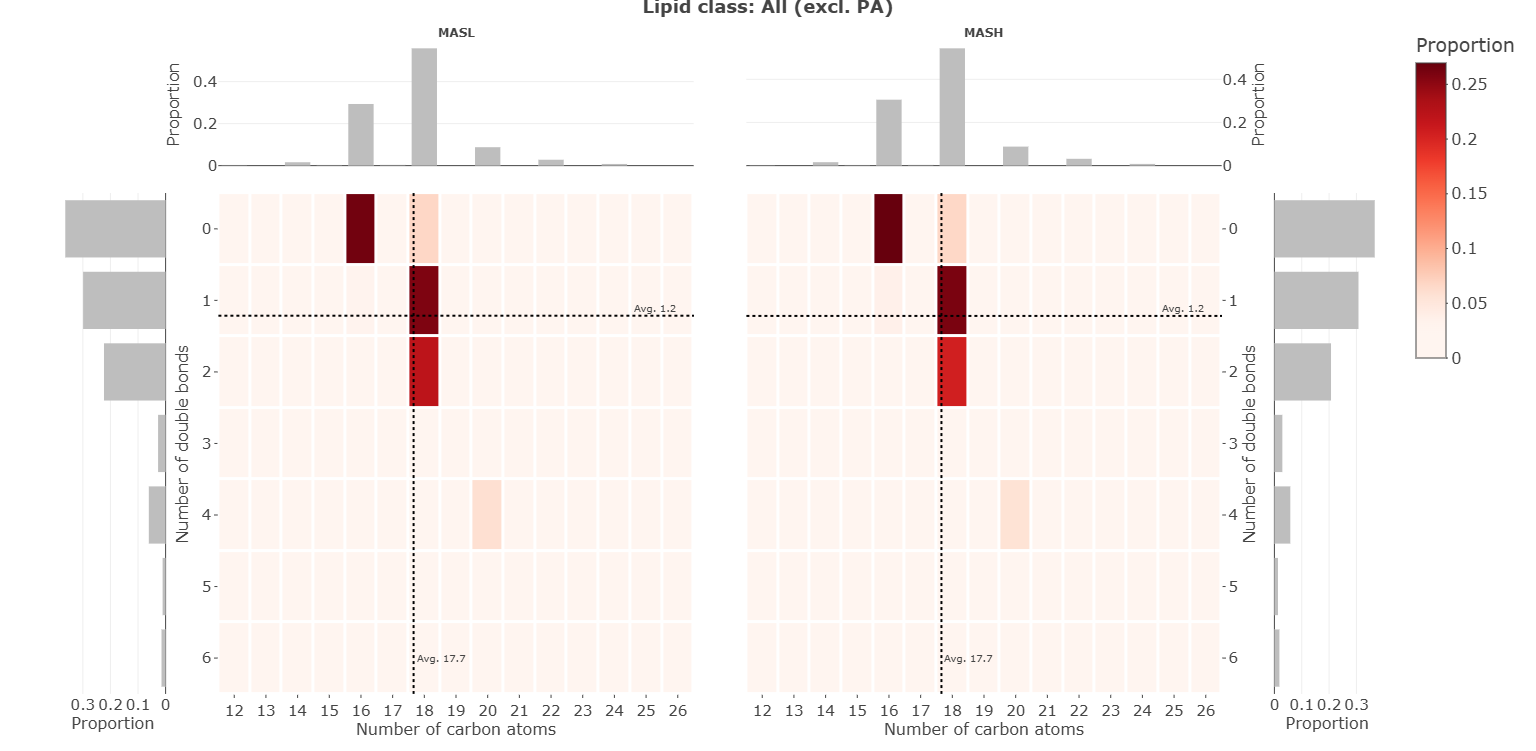


**Supplementary Figure 5.** Heatmap of the proportion of carbon atoms and double bond number of total fatty acid tail across all lipid classes between patients with MASL and MASH, including those using lipid-lowering drugs (n=86). The analysis was performed using iSODA.


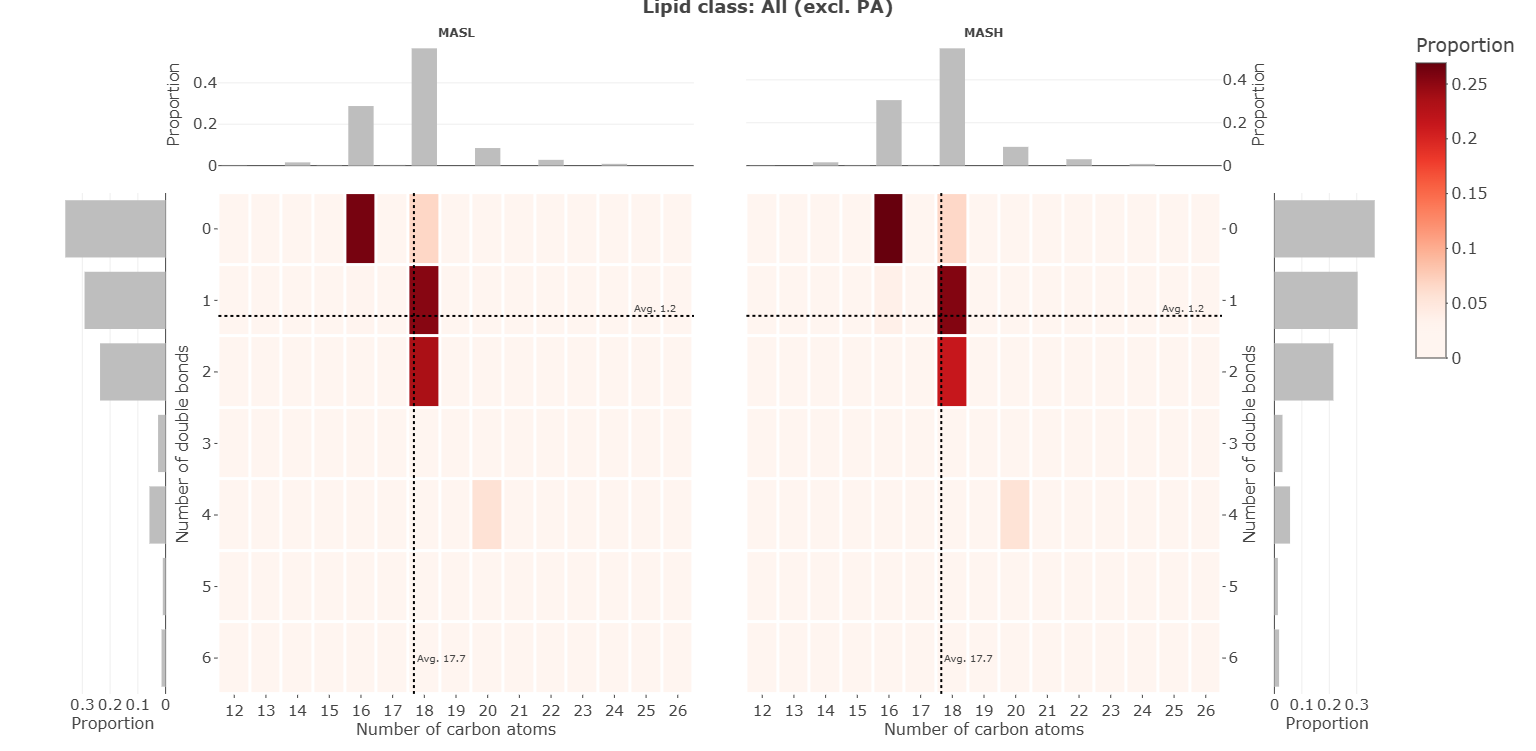


**Supplementary Figure 6.** Heatmap of the proportion of carbon atoms and double bond number of total fatty acid tail across all lipid classes between patients with MASL and MASH, excluding those using lipid-lowering drugs (n=56). The analysis was performed using iSODA.


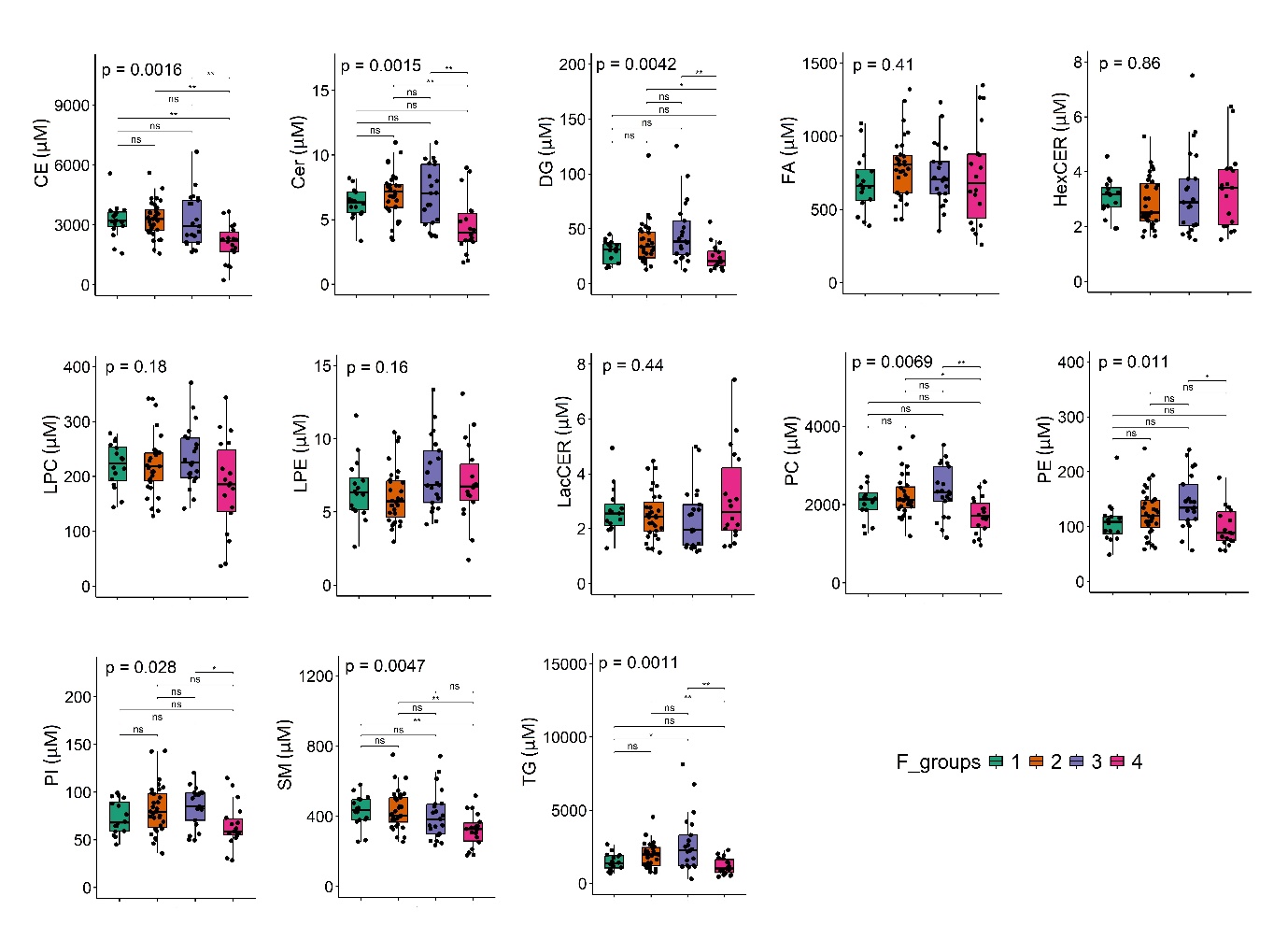


**Supplementary Figure 7.** Serum levels of 13 lipid classes between patients with fibrosis including those using lipid-lowering drugs (n=86). Fibrosis group 1 (F score 0-1), group 2 (F score 2), group 3 (F score 3), and group 4 (F score 4). Statistical analysis was done by Kruskal-Wallis, and in case where p-value < 0.05, Dunn’s post hoc test is conducted followed by Benjamini-Hochberg multiple testing correction. * p-value < 0.05, ** p-value < 0.01 between two groups.


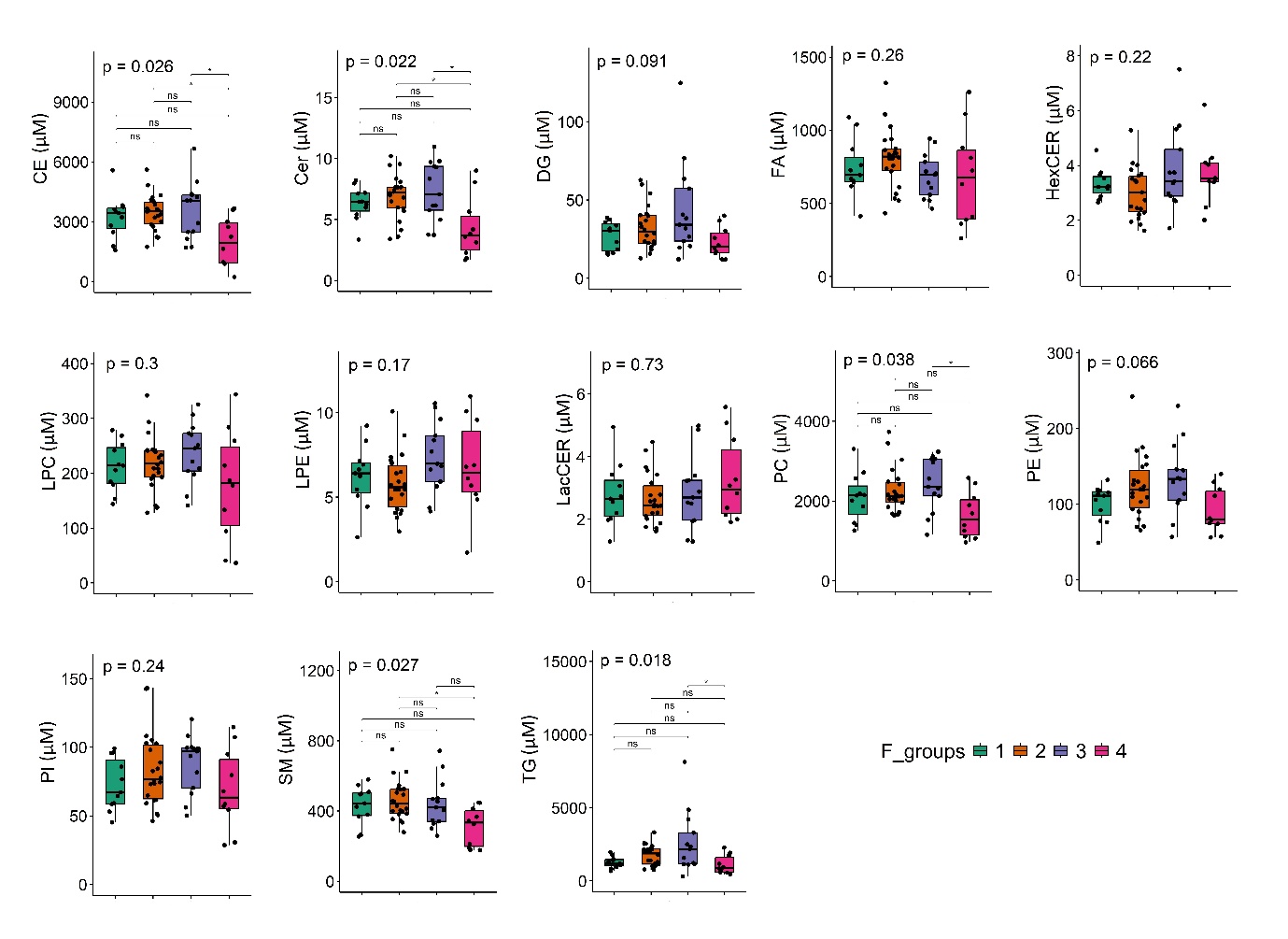


**Supplementary Figure 8.** Serum levels of 13 lipid classes between patients with fibrosis excluding those using lipid-lowering drugs (n=56). Fibrosis group 1 (F score 0-1), group 2 (F score 2), group 3 (F score 3), and group 4 (F score 4). Statistical analysis was done by Kruskal-Wallis, and in case where p-value < 0.05, Dunn’s post hoc test is conducted followed by Benjamini-Hochberg multiple testing correction. * p-value < 0.05 between two groups.

**Supplementary Table 1.** AUROCs of each candidate markers between patients with MASL and MASH, excluding those using lipid-lowering drugs. Logistic regression was performed between MASL and MASH using each candidate marker as a single predictor and in combination with ALT. Missing values on each observation were imputed using minimum value (in case where it shows no signal) and median (in case where it has interference during measurement). To validate the models a cross validation (100x) was done. Each time the data was split into a training set (75%) and a test set (25%). The split was stratified to ensure that the proportion of MASH cases is equal between the training and test set.

| **Candidate markers** | **AUROC (95% CI)**  (candidate) | **Validation**  **AUROC mean (95% CI)**  (candidate) | **AUROC (95% CI)**  (candidate+ALT) | **Validation**  **AUROC mean (95% CI)**  (candidate+ALT) |
| --- | --- | --- | --- | --- |
| ALT | 0.667 (0.475-0.858) | 0.703 (0.672-0.733) | - | - |
| Desmosterol | 0.750 (0.612-0.888) | 0.744 (0.717-0.771) | 0.789 (0.658-0.921) | 0.745 (0.719-0.771) |
| Cholesterol | 0.687 (0.508-0.866) | 0.696 (0.666-0.726) | 0.747 (0.570-0.923) | 0.730 (0.698-0.761) |
| CE | 0.687 (0.510-0.864) | 0.688 (0.657-0.719) | 0.719 (0.539-0.900) | 0.710 (0.676-0.744) |
| LPC | 0.689 (0.504-0.874) | 0.716 (0.689-0.742) | 0.719 (0.536-0.903) | 0.730 (0.700-0.761) |
| LPE | 0.704 (0.537-0.872) | 0.734 (0.705-0.764) | 0.711 (0.533-0.889) | 0.732 (0.701-0.763) |
| PC | 0.752 (0.584-0.919) | 0.746 (0.713-0.778) | 0.804 (0.644-0.965) | 0.793 (0.761-0.824) |
| PE | 0.709 (0.561-0.857) | 0.708 (0.678-0.738) | 0.736 (0.584-0.889) | 0.732 (0.703-0.760) |
| Phospholipid | 0.735 (0.563-0.906) | 0.732 (0.699-0.765) | 0.786 (0.619-0.952) | 0.779 (0.747-0.811) |
| CE 16:1 | 0.799 (0.633-0.966) | 0.786 (0.750-0.823) | 0.813 (0.657-0.969) | 0.785 (0.750-0.821) |
| PC 16:0_16:1 | 0.827 (0.696-0.957) | 0.811 (0.781-0.841) | 0.845 (0.728-0.962) | 0.821 (0.793-0.850) |
| PC 16:0_20:5 | 0.823 (0.709-0.937) | 0.825 (0.802-0.848) | 0.820 (0.691-0.948) | 0.814 (0.791-0.837) |
| PC 18:0_20:2 | 0.829 (0.691-0.967) | 0.809 (0.782-0.837) | 0.829 (0.679-0.979) | 0.805 (0.775-0.834) |
| PC 18:0_20:5 | 0.825 (0.712-0.938) | 0.830 (0.807-0.852) | 0.815 (0.684-0.945) | 0.811 (0.786-0.835) |
| TG 54:7_FA 16:1 | 0.803 (0.669-0.936) | 0.795 (0.768-0.823) | 0.816 (0.681-0.952) | 0.791 (0.764-0.818) |
| TG 54:7_FA 22:6 | 0.798 (0.664-0.932) | 0.787 (0.759-0.815) | 0.813 (0.679-0.947) | 0.774 (0.744-0.804) |
| TG 58:6_FA 16:0 | 0.798 (0.662-0.933) | 0.778 (0.752-0.804) | 0.798 (0.652-0.943) | 0.761 (0.733-0.790) |
| TG 60:12_FA 22:6 | 0.763 (0.604-0.921) | 0.763 (0.734-0.793) | 0.810 (0.662-0.957) | 0.788 (0.758-0.817) |

**Supplementary Table 2.** AUROCs of any combinations of candidate markers between patients with MASL and MASH, excluding those using lipid-lowering drugs. Logistic regression was performed between MASL and MASH using any possible combinations with or without ALT. The combinations were selected by considering any low mutual correlations between candidate markers. Missing values on each observation were imputed using minimum value (in case where it shows no signal). To validate the models a cross validation (100x) was done. Each time the data was split into a training set (75%) and a test set (25%). The split was stratified to ensure that the proportion of MASH cases is equal between the training and test set.

| **Lipid candidates** | **AUROC (95% CI)**  Lipid combination | **Validation**  **AUROC mean (95% CI)**  (candidate) | **AUROC (95% CI)**  Lipid combination + ALT | **Validation**  **AUROC mean (95% CI)**  (candidate+ALT) |
| --- | --- | --- | --- | --- |
| CE 16:1 + TG 60:12_FA22:6 | 0.871 (0.758-0.983) | 0.828 (0.800-0.856) | 0.901 (0.812-0.991) | 0.853 (0.828-0.878) |
| PC 16:0_16:1 + PC 16:0_20.5 | 0.861 (0.745-0.976) | 0.844 (0.818-0.869) | 0.886 (0.790-0.982) | 0.861 (0.838-0.884) |
| PC 16:0_16:1 + PC 18:0_20:5 | 0.862 (0.746-0.979) | 0.844 (0.815-0.872) | 0.864 (0.752-0.975) | 0.843 (0.813-0.873) |
| PC 16:0_16:1 + TG 54:7_FA22:6 | 0.872 (0.765-0.980) | 0.844 (0.818-0.870) | 0.888 (0.790-0.986) | 0.846 (0.820-0.873) |
| PC 16:0_16:1 + TG 58:6_FA16:0 | 0.869 (0.763-0.975) | 0.823 (0.794-0.851) | 0.861 (0.752-0.969) | 0.810 (0.782-0.839) |
| PC 16:0_16:1 + TG 60:12_FA22:6 | 0.889 (0.788-0.991) | 0.863 (0.840-0.887) | 0.910 (0.820-1.000) | 0.873 (0.849-0.897) |
| PC 18:0_20:2 + TG 60:12_FA22:6 | 0.888 (0.798-0.978) | 0.851 (0.830-0.871) | 0.896 (0.800-0.993) | 0.837 (0.812-0.862) |

**
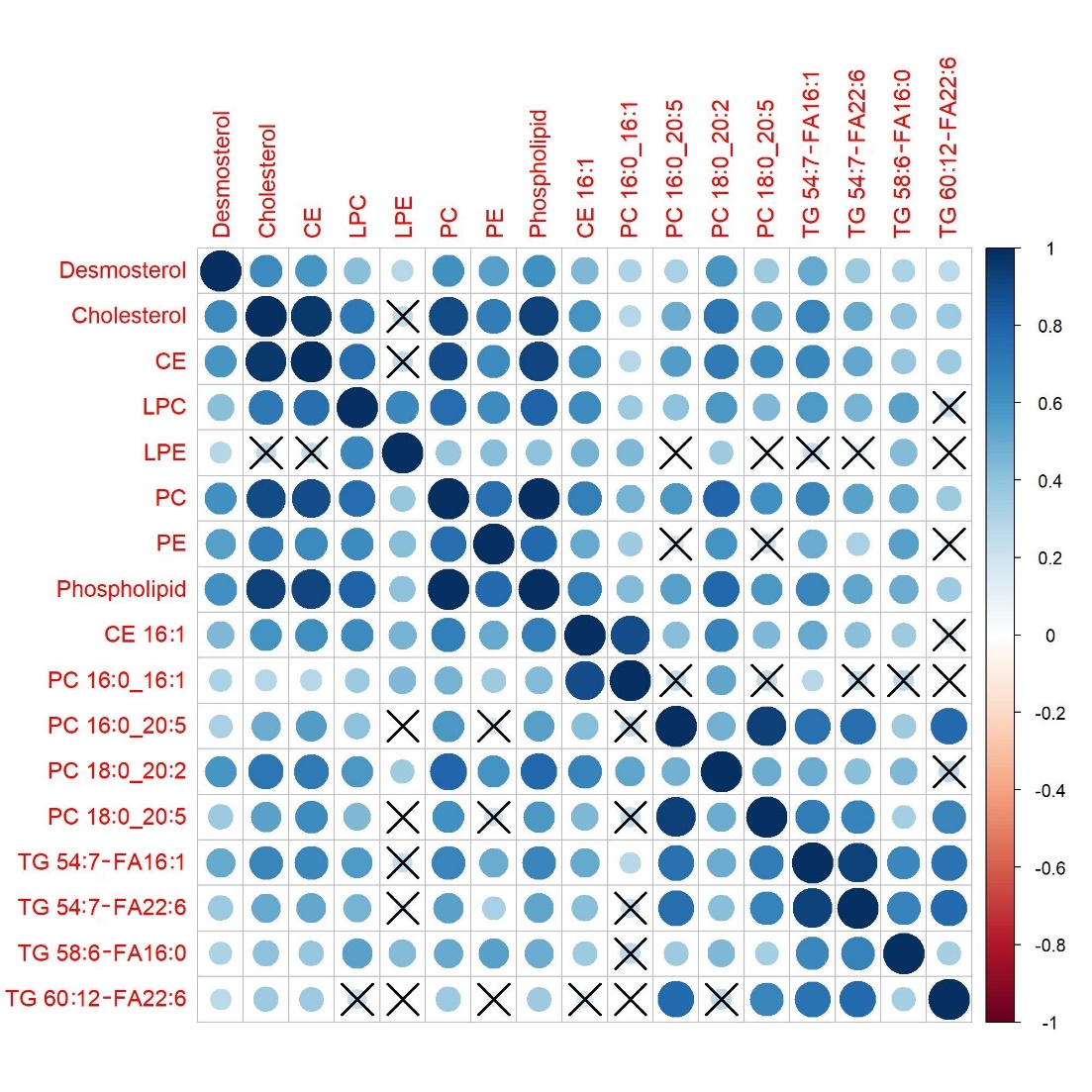
**

**Supplementary Figure 9.** Correlation heatmap between each candidate markers in the patients with MASLD, excluding those using lipid lowering drugs. Analysis was conducted by Pearson correlation. Missing values on each observation were imputed using minimum value (in case where it shows no signal) and median (in case where it has interference during measurement). Color intensity is proportional to the correlation coefficient (r) while the cross sign is showing insignificant correlation (p-value > 0.05).


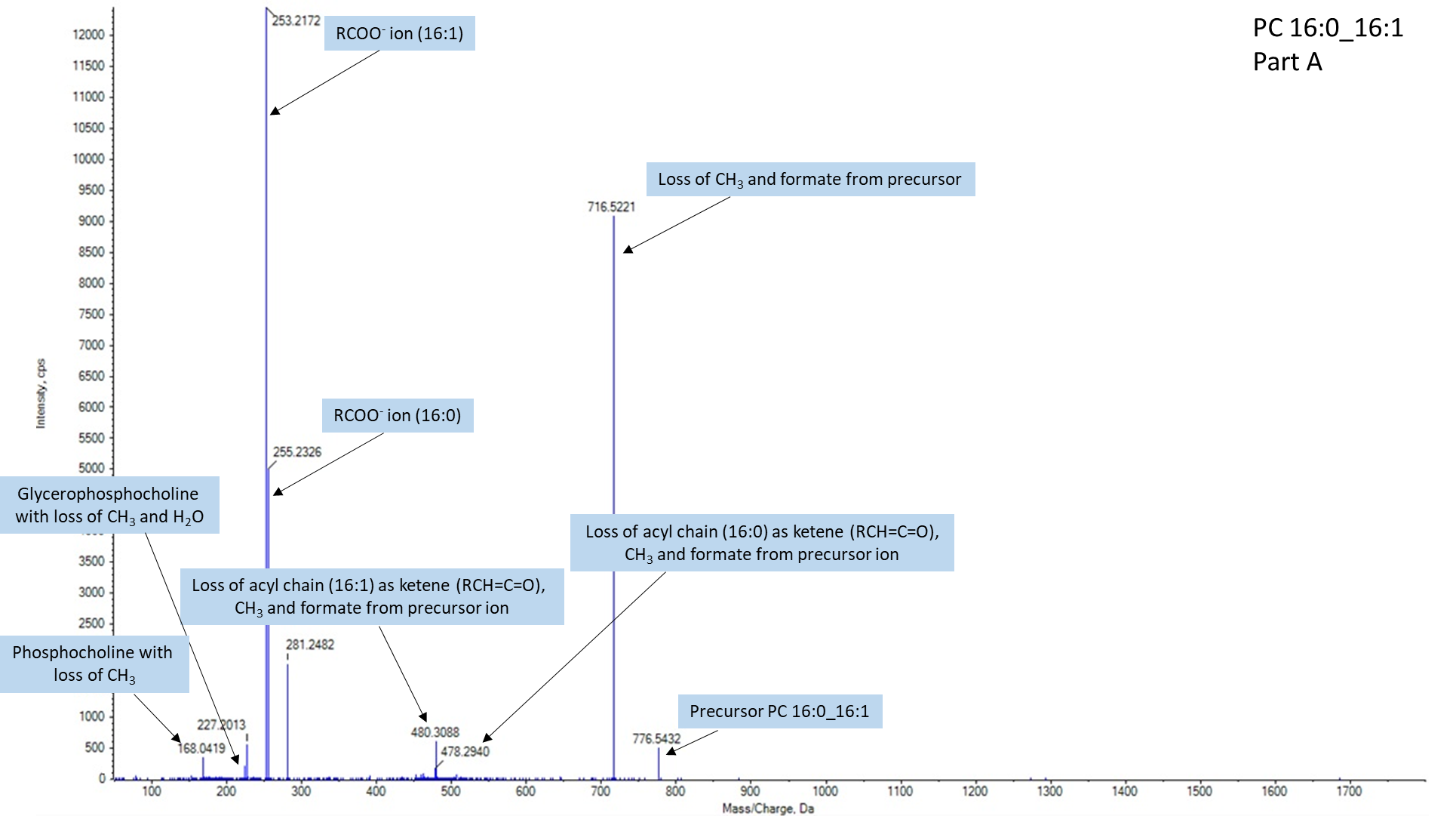


**A**


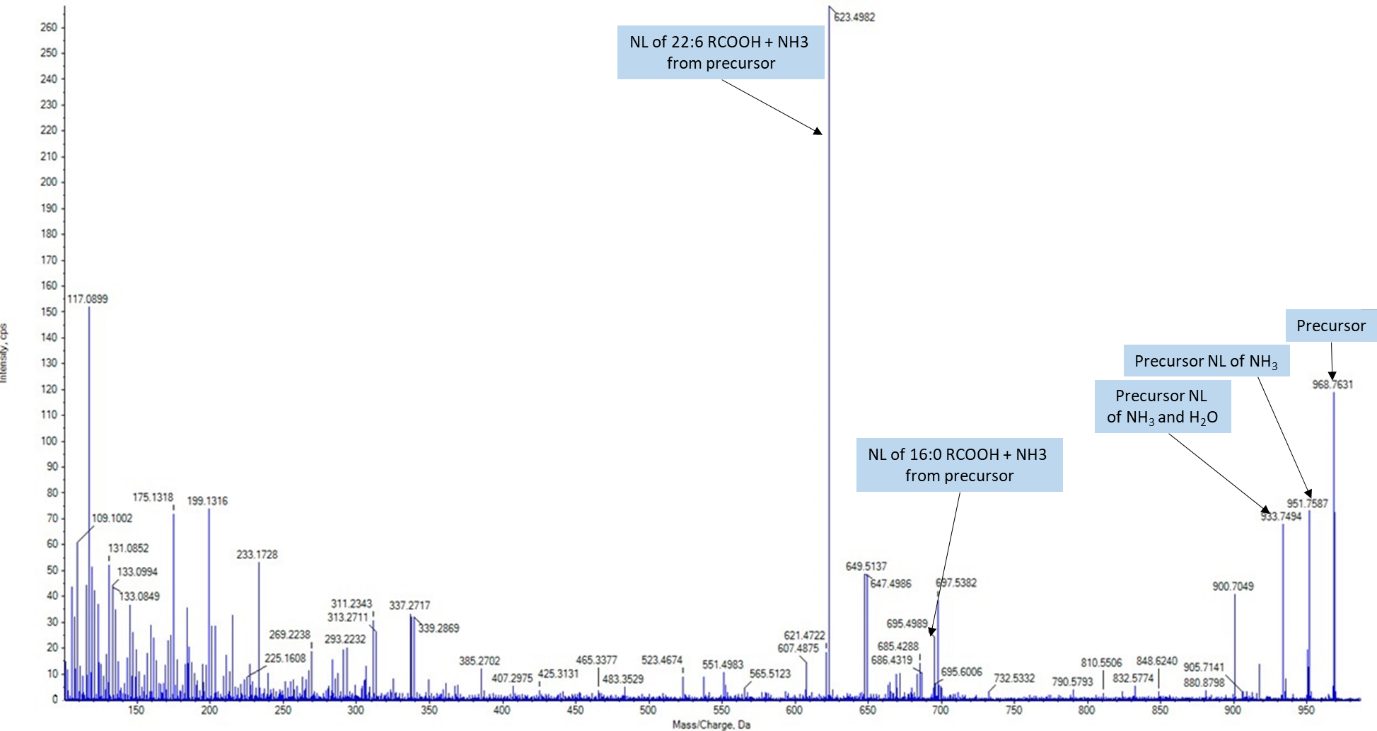


**B**

**Supplementary Figure 10.** MS/MS spectra of PC 16:0_16:1 - precursor [M+HCOO]^-^ (A) ^-^ and TG 60:12-FA22:6 - precursor [M+NH4]^+^ (B)

**
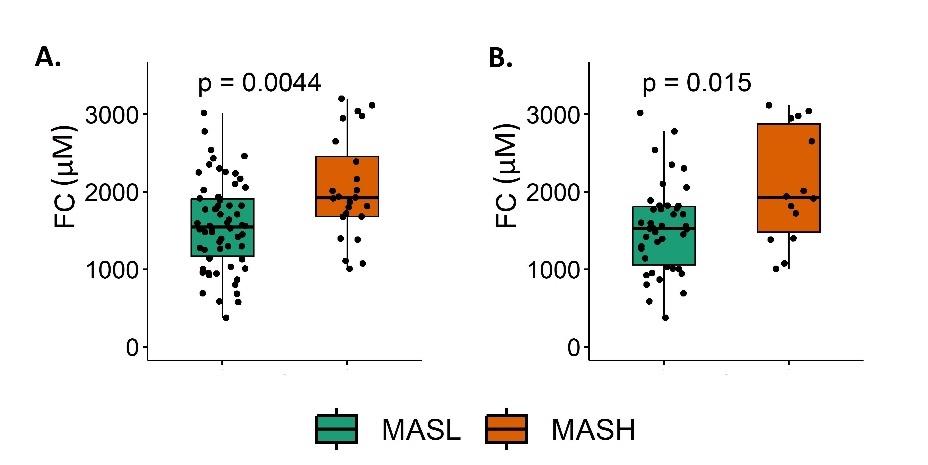
**

**Supplementary Figure 11.** Estimated serum free cholesterol (FC) level between patients with MASL and MASH including those using lipid lowering drugs (n=86) (A) and excluding those using lipid lowering drugs (n=56) (B). Statistical analysis was performed by Wilcoxon test. The FC fraction was estimated by subtracting the cholesterol ester fraction from the total cholesterol analysis result.

**Supplementary Table 3.** Correlation analysis of each candidate marker vs each histological feature between patients with MASLD excluding those using lipid lowering drugs. Analysis was conducted by Spearman correlation.

| **Lipid** | **Histopathological features (r, p-value)** | | | |
| --- | --- | --- | --- | --- |
|  | **Steatosis** | **Lobular inflammation** | **Ballooning** | **NAS** |
| Desmosterol | 0.299, 0.028 | 0.237, 0.085 | 0.403, 0.002 | 0.428, 0.001 |
| Cholesterol | 0.335, 0.012 | 0.136, 0.319 | 0.079, 0.562 | 0.266, 0.047 |
| CE | 0.302, 0.024 | 0.094, 0.489 | 0.102, 0.456 | 0.248, 0.066 |
| LPC | 0.300, 0.025 | 0.149, 0.272 | 0.252, 0.060 | 0.314, 0.018 |
| LPE | 0.234, 0.083 | 0.182, 0.180 | 0.362, 0.006 | 0.340, 0.010 |
| PC | 0.365, 0.006 | 0.092, 0.499 | 0.159, 0.242 | 0.321, 0.016 |
| PE | 0.290, 0.030 | 0.217, 0.109 | 0.162, 0.232 | 0.305 0.022 |
| Phospholipid | 0.344, 0.009 | 0.106, 0.437 | 0.171, 0.209 | 0.317, 0.017 |
| CE 16:1 | 0.498, <0.001 | 0.204, 0.132 | 0.303, 0.023 | 0.499, <0.001 |
| PC 16:0_16:1 | 0.454, <0.001 | 0.305, 0.022 | 0.514, <0.001 | 0.618, <0.001 |
| PC 16:0_20:5 | 0.252, 0.061 | 0.096, 0.481 | 0.068, 0.617 | 0.252, 0.061 |
| PC 18:0_20:2 | 0.415, 0.003 | 0.236, 0.102 | 0.301, 0.035 | 0.474, <0.001 |
| PC 18:0_20:5 | 0.270, 0.048 | 0.005, 0.971 | 0.124, 0.370 | 0.276, 0.043 |
| TG 54:7_FA 16:1 | 0.352, 0.008 | 0.137, 0.314 | 0.204, 0.132 | 0.371, 0.005 |
| TG 54:7_FA 22:6 | 0.342, 0.010 | 0.158, 0.244 | 0.188, 0.166 | 0.368, 0.005 |
| TG 58:6_FA 16:0 | 0.394, 0.003 | 0.219, 0.105 | 0.249, 0.064 | 0.423, 0.001 |
| TG 60:12_FA 22:6 | 0.246, 0.070 | 0.222, 0.104 | 0.105, 0.445 | 0.296, 0.028 |


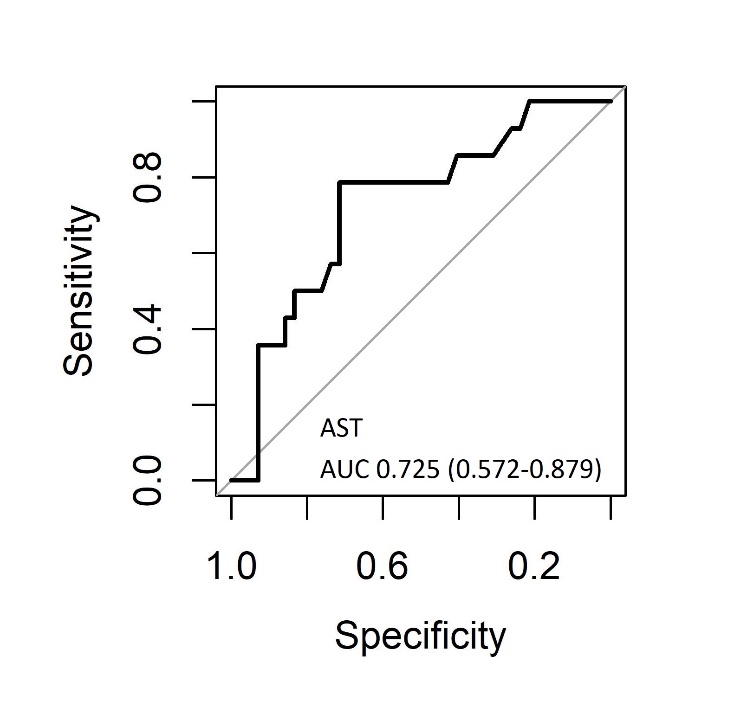


**Supplementary Figure 12.** ROC curve of AST
